# Effect of Printing Orientation and Post-Curing Time on the Mechanical Properties of 3D-Printed Denture Base Resin

**DOI:** 10.3390/jfb17010001

**Published:** 2025-12-19

**Authors:** Ivet Dzhondrova, Ilia Liondev, Iva Taneva, Todor Bogdanov, Todor Uzunov, Dimitar Kirov

**Affiliations:** 1 Department of Prosthetic Dental Medicine, Faculty of Dental Medicine, Medical University of Sofia, 1000 Sofia, Bulgaria; i.lyondev@fdm.mu-sofia.bg (I.L.); i.taneva@fdm.mu-sofia.bg (I.T.); uzunov@fdm.mu-sofia.bg (T.U.); d.kirov@fdm.mu-sofia.bg (D.K.); 2 Department of Physics and Biophysics, Medical University of Sofia, 1000 Sofia, Bulgaria

**Keywords:** additive manufacturing, CAD/CAM, photopolymer resin, build angle, post-polymerization, flexural strength, microhardness

## Abstract

Additive manufacturing is increasingly integrated into dental technology, yet the mechanical performance of 3D-printed denture base resins remains strongly influenced by printing orientation and post-curing duration. This study evaluated the combined effect of three printing orientations (0°, 45°, 90°) and three post-curing times (30, 45, 60 min) on the flexural strength and surface microhardness of a denture base resin. Specimens designed in Blender and fabricated using NextDent Denture 3D+ resin were subjected to three-point bending tests (*n* = 5 per group) and Vickers microhardness measurements (*n* = 10 per group). One-way ANOVA assessed main and interaction effects. Printing orientation had a significant influence on flexural strength, with horizontally printed specimens exhibiting the highest values, whereas vertically printed specimens were consistently weaker. Post-curing time did not significantly affect flexural strength within any orientation. In contrast, microhardness increased progressively with longer post-curing durations, regardless of orientation, indicating continued surface polymerisation. Because flexural strength and hardness responded differently to curing duration, no single post-curing time was universally optimal; however, 0° printing consistently produced the strongest specimens for this resin–printer system. This trade-off is clinically relevant, because dentures require high flexural strength to resist fracture and sufficient hardness to minimise wear.

## 1. Introduction

Additive manufacturing has become an integral part of current dental-technology workflows, enabling the rapid and cost-effective fabrication of complete and partial dentures that once required multiple clinical appointments and highly skilled laboratory work [1,2,3]. Among current additive technologies, stereolithography (SLA) is particularly advantageous for denture-base fabrication because it provides high dimensional accuracy, excellent surface reproduction and minimal material waste [4,5]. Recent studies further indicate that SLA-fabricated dentures can achieve patient satisfaction comparable to conventional heat-polymerised PMMA while reducing overall production time and number of appointments [6]. Unlike heat-cured PMMA, however, the photopolymer resins used in SLA printing are delivered in a partially reacted state; therefore, both the selected build parameters and the subsequent post-curing cycle play a decisive role in achieving optimal polymer conversion and mechanical performance [7].

Mechanical performance is critical for denture longevity, as denture bases are subjected to complex combinations of compressive, tensile, flexural and shear stresses during mastication. Insufficient strength or elasticity can lead to deformation, material fatigue or midline fractures, which are among the most common clinical failures. Although occlusal wear primarily affects denture teeth rather than the denture base, evaluating the Vickers hardness of denture-base resins remains clinically relevant. Hardness provides an indirect measure of the degree of polymerisation and cross-link density, both of which contribute to long-term surface stability, resistance to microabrasion during hygiene procedures, and overall durability of the intaglio and polished surfaces. Moreover, hardness frequently correlates with flexural strength and fracture resistance, making it a meaningful indicator of material performance in additively manufactured dentures [8,9].

Because the properties of printed photopolymers are strongly technique-sensitive, both the printing process and the post-curing stage substantially influence the final mechanical behaviour of printed dentures [10]. Altarazi et al. categorised the determinants of printed dentures quality into uncontrollable factors—such as resin chemistry, light power and wavelength—and controllable factors including build orientation, curing temperature and exposure time [11]. While earlier studies focused primarily on interim or diagnostic applications of printed dentures, emerging evidence demonstrates that evolving resin formulations may offer increasingly reliable mechanical properties suitable for long-term use [12].

Build orientation is particularly influential because it dictates how layers are stacked and supported during polymerisation, thus affecting internal stress distribution and interlayer cohesion. Horizontal (0°) printing often produces smoother intaglio surfaces and favourable interlayer fusion, whereas vertical (90°) printing may increase unsupported areas and stair-stepping effects [13,14]. These microstructural differences can lead to variations in flexural strength and surface hardness—properties that are essential for removable dentures that endure cyclic loading in the oral cavity [15]. Practical considerations also vary: 0° builds minimise production time but require more support material, 90° builds reduce material usage at the expense of printing time, and 45° builds frequently provide high dimensional accuracy but may exhibit increased surface roughness [16,17,18].

Post-curing further advances polymerisation by activating residual methacrylate groups, increasing cross-link density and reducing the concentration of unreacted monomer [19,20]. The effectiveness of this process depends on spectral compatibility between the curing unit and the photoinitiator, as well as temperature, oxygen inhibition and object geometry. Although extending post-curing generally enhances mechanical properties and biocompatibility, excessive exposure may induce dimensional distortion or colour change [21]. Prior studies also report material-specific variation: Aati et al. [19] observed improved flexural properties with extended curing, whereas Altarazi et al. [11] found diminishing returns beyond 30 min. The three post-curing times selected in this study (30, 45 and 60 min) were chosen to capture the recommended duration from the manufacturer as well as clinically relevant extensions commonly used in dental laboratories.

Despite increasing interest in printed denture-base resins, existing research often examines a narrow range of orientations or a single post-curing protocol, making direct comparisons difficult. Far fewer studies have investigated the combined influence of these variables or assessed key functional properties such as Vickers hardness—an indicator of wear resistance—and flexural strength, a primary predictor of fracture. Moreover, most available data derive from static in vitro testing that does not reflect intraoral conditions, such as thermocycling, cyclic loading or microbial colonisation. For instance, Shim et al. [12] demonstrated that build orientation can even influence biofilm accumulation, with 0° specimens exhibiting greater Candida albicans adhesion. These gaps highlight the need for more comprehensive investigations that systematically examine both orientation and curing protocols.

The present study therefore aimed to evaluate the effect of three printing orientations (0°, 45°, 90°) and three post-curing times (30, 45 and 60 min) on the Vickers microhardness and flexural strength of a 3D-printed denture-base resin. The null hypothesis was that neither post-curing time nor printing orientation would significantly influence the mechanical properties of the evaluated material.

## 2. Materials and Methods

### 2.1. Study Design

**Figure 1:**
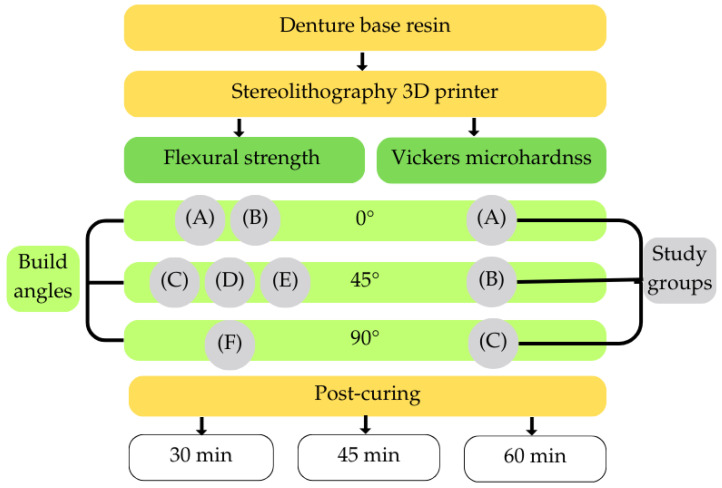
Overview of the experimental workflow for evaluating the effects of printing orientation and post-curing duration on the mechanical properties of a 3D-printed denture base resin. Flexural strength (FS) and Vickers microhardness (VHN) tests were conducted on specimens printed at three build angles (0°, 45°, 90°). For flexural testing, six orientation–support configurations (Groups A–F) were produced, while three groups (A–C) were prepared for hardness testing. All specimens underwent post-curing for 30, 45, or 60 min prior to mechanical evaluation.

This laboratory-based quantitative study aimed to evaluate the influence of printing orientation and post-curing duration on the flexural strength and Vickers microhardness of a denture base resin fabricated using stereolithography (SLA) 3D printing (Figure 1).

### 2.2. Grouping

All specimens were divided into nine primary experimental groups, representing the combinations of three printing orientations (0°, 45°, and 90°) and three post-curing durations (30, 45, and 60 min). For the flexural strength testing the 0° orientation included two distinct specimen positions, while the 45° orientation included three configurations, each differing in rotational alignment and support placement. The three orientations tested in this study (0°, 45°, 90°) were selected because they represent the most commonly recommended angulations in the literature and reflect practical extremes and midpoints used in clinical laboratory settings. The group printed at 0° and post-cured for 30 min served as the control group, as these parameters are aligned with the manufacturer’s standard recommendations for the material and printer used.

### 2.3. Specimen Preparation

Rectangular bar-shaped specimens for flexural testing were digitally designed according to ISO 20795-1 [22], with nominal dimensions of 64 × 10 × 3.3 mm. Disc-shaped specimens for Vickers hardness testing with nominal dimensions of 20 mm × 3 mm. After printing and post-polymerisation, the actual specimen dimensions were measured using a digital calliper with 0.01 mm resolution to verify dimensional accuracy. This dimensional control step ensured that deviations from the digital design remained within clinically acceptable tolerances.

Test specimens were digitally designed using Blender v2.73 (Blender Foundation, Amsterdam, The Netherlands). The digital workflow allowed for precise control over specimen geometry and ensured uniformity across all experimental groups. Once the designs were finalised, they were exported for fabrication and printed using a Pro S, NextDent stereolithography 3D printer (3D Systems, Soesterberg, The Netherlands), following the manufacturer’s recommended parameters for optimal accuracy and material performance. The printing material used in this study was NextDent Denture 3D+ light-cured resin (3D Systems, Soesterberg, The Netherlands), a photopolymer specifically formulated for denture-base applications and engineered to provide consistent curing behaviour within SLA environments.

Following the printing stage, all specimens underwent a standardised post-processing protocol to eliminate uncured resin and ensure full polymer conversion. The printed samples were first cleaned in 95% isopropyl alcohol using an ultrasonic bath (Pro Wash, 3D Systems, Soesterberg, The Netherlands), which facilitates the removal of residual resin from the surface and internal layer interfaces. After cleaning, the specimens were subjected to ultraviolet post-curing in a Pro Cure photopolymerization oven (3D Systems, Soesterberg, The Netherlands). Post-curing was performed at a controlled temperature of 80 °C with a wavelength of 405 nm for the designated durations assigned to each group. This step was critical for completing polymer cross-linking and achieving the final mechanical properties required for denture-base materials.

**Figure 2:**
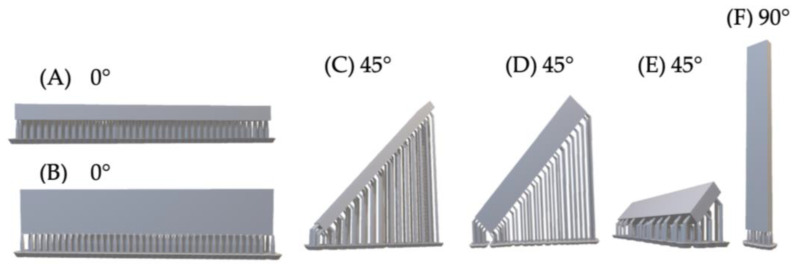
CAD design of rectangular bar specimens for the three-point bending test according to ISO 20795-1:2013. (**A**,**B**) 0° orientation (horizontal): Specimens printed flat, with two alternative orientations of the tested surface relative to the build platform. (**C**–**E**) 45° orientation (angled): Specimens printed at a 45° tilt, illustrating the three rotational positions used to vary layer deposition and support placement. (**F**) 90° orientation (vertical): Specimen printed upright, with layers deposited along the height.

For flexural strength testing a total of 90 specimens were produced and subsequently divided into 18 subgroups based on the combined variables of build orientation and post-curing time (*n* = 5 per group). This experimental design allowed for a systematic assessment of the individual and interactive effects of printing orientation and post-curing duration. The specific build-orientation configurations are illustrated in Figure 2.

Although both Groups A and B were printed at a nominal 0° orientation, they differed in the orientation of the tested surface relative to the build platform. In Group A, the tested surface was positioned parallel to the platform, and layers were deposited through the thickness of the specimen. Supports were attached to the opposite, non-tested surface. In Group B, the tested surface was positioned perpendicular to the platform, resulting in layers being deposited across the width of the specimen. In this configuration, supports were attached to the side of the bar. Although both positions are classified as 0°, the markedly different layer-stacking directions and support-contact regions justified treating these as separate experimental groups.

Groups C, D, and E were all printed at a 45° build angle but differed in the rotational orientation of the rectangular specimens around their long axis. This resulted in three distinct tested-surface orientations and corresponding differences in support placement and layer-deposition direction. Because these geometric differences can affect polymerization stresses and mechanical performance, each configuration was treated as a separate subgroup.

In Group F (90° orientation), specimens were printed vertically with the tested surface aligned perpendicular to the platform. Layers were deposited along the height of the specimen, and supports were attached only to the bottom edge.

Each build-orientation group was subsequently subdivided into three post-curing subgroups corresponding to curing durations of 30, 45, and 60 min. This experimental structure enabled a systematic evaluation of the individual and combined effects of build angle and post-curing time on the mechanical performance of the printed material.

**Figure 3:**
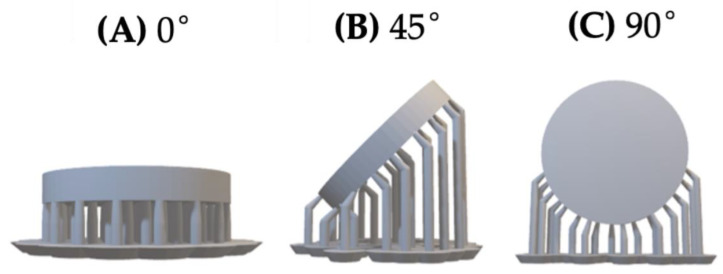
CAD design of disc-shaped specimens for the Vickers microhardness test with nominal dimensions of 20 mm × 3 mm (**A**) 0° orientation (horizontal): Specimen printed flat on the build platform. (**B**) 45° orientation (angled): Specimen printed at a 45° tilt to modify layer deposition and support placement. (**C**) 90° orientation (vertical): Specimen printed upright, with layers deposited along the height.

For the evaluation of surface microhardness, a total of 90 disc-shaped specimens were fabricated. These specimens (*n* = 10 per group) were distributed into nine experimental groups based on the combination of three build orientations (0°, 45°, and 90°) and three post-curing durations (30, 45, and 60 min). This mirrored the grouping structure used for flexural strength testing, allowing for direct comparison between the two mechanical tests. The specific build-orientation configurations used for microhardness evaluation are depicted in Figure 3.

The tested surface was the surface without supports (for groups A and B), ensuring that no support-removal defects or residual attachment marks influenced the indentation results. Before hardness testing, the tested surfaces were wet-polished sequentially using 600-, 800-, and 1200-grit silicon-carbide (SiC) abrasive papers under continuous water irrigation. All specimens were then rinsed with distilled water, ultrasonically cleaned for 5 min, and air-dried.

### 2.4. Flexural Strength Testing

Flexural testing was conducted using a MultiTest 2.5-i Tensile and Compression Test System (Mecmesin Ltd., Slinfold, West Sussex, UK) equipped with a standard three-point bending fixture. Testing followed ISO 20795-1:2013, with a 50 mm support span. The crosshead was initially lowered at 250 mm/min until a contact force of 10 N was reached, then reduced to 2 mm/min to stabilise the specimen at 2.5 N. After a 0.25 mm displacement, the loading rate was set to 5 mm/min and maintained until fracture (Fmax) or a maximum load of 1000 N. Flexural strength (FS) was automatically calculated by the Emperor™(version 1.18) software using the formula FS = 3FL/2bh^2^, where F is the maximum load, L the support span, b the specimen width, and h its thickness.

### 2.5. Microhardness Testing

Hardness measurements were performed using a Zwick Roell ZHVμ Micro Vickers hardness tester (Indentec Hardness Testing Machines Ltd., Brierley Hill, UK), a precision instrument widely used for evaluating the surface mechanical properties of polymeric dental materials. The device utilises a square-based diamond pyramid indenter with an angle of 136° between opposing faces, providing a standardised means of generating controlled surface impressions. For each measurement, a load of 0.5 kgf (HV0.5) was applied for a dwell time of 10 s to ensure adequate penetration and minimise elastic recovery effects. Following indentation, the two diagonals of each impression were measured at 20× magnification in both horizontal and vertical directions, and the average of these two values was used to calculate the final Vickers hardness number for each specimen.

### 2.6. Statistical Analysis

All statistical analyses were conducted using IBM SPSS Statistics for Windows, Version 24 (IBM Corp., Armonk, NY, USA). The normality of the data distribution was assessed using the Shapiro–Wilk test, while the homogeneity of variances across groups was evaluated using Levene’s test. After confirming that the data met the necessary parametric assumptions, comparisons between groups were performed using one-way ANOVA to determine the independent effects of printing orientation and post-curing duration. When significant differences were detected, Tukey’s post hoc test was applied to identify pairwise differences among groups. Effect sizes for one-way ANOVA were calculated using eta squared (η^2^) and are reported to quantify the magnitude of group differences. A significance level of *p* ≤ 0.05 was set for all analyses.

## 3. Results

The flexural strength of the specimens was predominantly influenced by the printing orientation, while the contribution of post-curing time appeared minimal (Table 1). Both Groups A and B were fabricated at a 0° orientation, although their tested surfaces were positioned differently during printing—Group A with the tested surface parallel to the build platform, and Group B with the tested surface oriented perpendicular to the platform. This distinction resulted in notable variation in mechanical performance. The substantial difference between Groups A and B can be attributed to their layer-stacking directions. In Group A, the tested surface faced upward, resulting in supports placed only on the opposite, non-tested surface and in layers deposited through the thickness of the bar. This configuration minimises surface defects and reduces stress concentrations. In contrast, Group B required supports to be attached along the side of the specimen, and layers were deposited across the width. Side-support attachment increases the likelihood of localised polymerization shrinkage, leading to lower flexural strength values. This quantitative influence of surface orientation and support geometry is reflected in the markedly different mean strengths observed between the two 0° groups. Among all experimental groups, specimens printed at 0° (Group A) consistently demonstrated the highest flexural strength values. At a post-curing time of 30 min, Group A achieved a maximum individual strength of 246.6 MPa and a mean value of 213.26 ± 22.25 MPa. In contrast, the lowest flexural strength recorded in the study occurred in Group B (also printed at 0°), post-cured for 45 min, with a minimum value of 98.1 MPa and a mean of 133.4 ± 20.02 MPa. One-way ANOVA showed large effect sizes for printing orientation at all curing times, with η^2^ values ranging from 0.69 to 0.85, indicating that orientation accounted for the majority of variance in flexural strength. In contrast, effect sizes for post-curing time within individual orientation groups were small to moderate (η^2^ ≤ 0.25).

**Figure 4:**
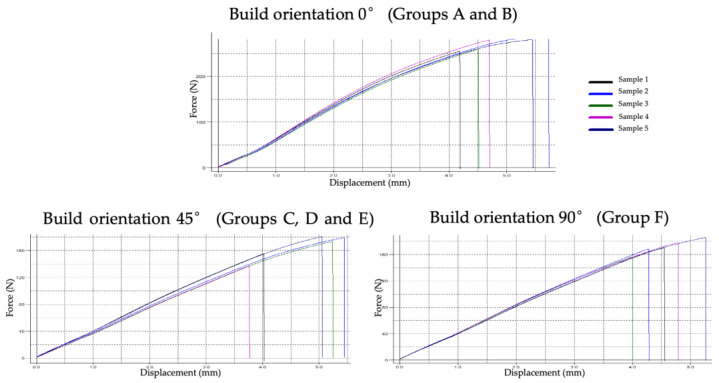
Representative load–deflection curves for specimens printed at 0°, 45°, and 90° orientations, generated by the testing software. These curves illustrate the typical mechanical response of each build orientation under three-point bending and complement the flexural strength values presented in Table 1.

**Figure 5:**
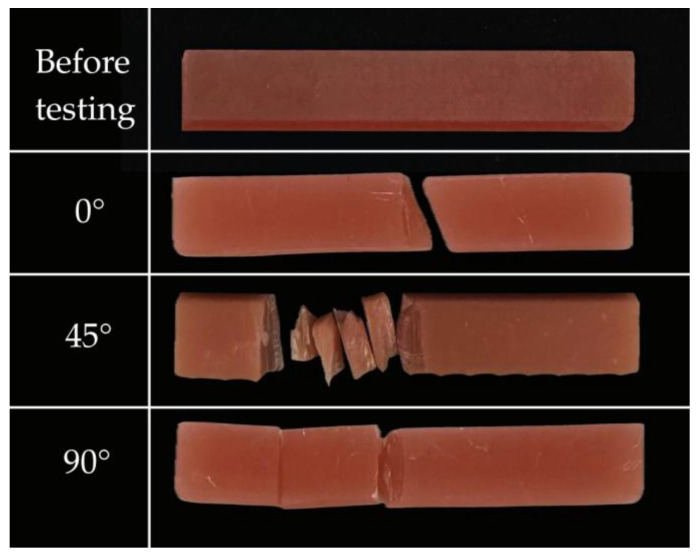
Representative pre- and post-fracture appearance of the tested 3D-printed specimens subjected to three-point bending (ISO 20795-1). The first image shows an intact, untested specimen. The remaining images display typical fracture appearances for specimens printed at 0°, 45°, and 90° orientations.

Across the remaining groups (C through F), representing the 45° and 90° orientations, flexural strength values ranged from 134.98 to 166.16 MPa. No statistically significant differences were identified among these groups, indicating that intermediate and vertical orientations produced comparable performance. Although slight increases in strength were observed with extended post-curing times—such as the rise to 227.58 ± 11.33 MPa for the 0° group after 60 min—these changes did not reach statistical significance within any orientation category. To illustrate the typical mechanical behaviour of each build orientation, three representative groups from the three build orientations (0°, 45°, and 90°) were selected for generating load–deflection curves using the testing software (Figure 4). Representative pre- and post-fracture appearances of the tested specimens following three-point bending are shown in Figure 5, illustrating the intact specimens prior to testing and the typical fracture patterns observed in samples printed at 0°, 45°, and 90° orientations.

The Vickers hardness of the 3D-printed denture base resin was significantly affected by both printing orientation and post-curing duration (Table 2). Across all curing times, specimens produced at the 0° orientation (Group A) demonstrated the highest hardness values, while those fabricated at 45° (Group B) consistently exhibited the lowest. This trend was already evident at the 30-min curing interval, during which the 0° group showed a mean hardness of 28.5 ± 3.32 HV, compared with 16.0 ± 1.34 HV for the 45° group and 20.9 ± 1.37 HV for the 90° group.

Prolonging the post-curing time produced additional improvements in hardness, particularly for the 0° orientation. After 60 min of curing, the mean hardness of the 0° specimens increased to 34.0 ± 3.40 HV, representing a statistically significant enhancement relative to the 30-min condition (*p* < 0.05). In contrast, no statistically significant changes were observed within the 90° group across the different curing durations, indicating that the effect of prolonged curing was minimal for vertically printed specimens. The 45° group demonstrated modest increases in hardness with extended curing; however, its values remained significantly lower than those of the 0° orientation at every time point examined. For Vickers hardness, printing orientation exhibited very large effects across all curing times (η^2^ = 0.81–0.88), explaining over 80% of the variance. The effect of post-curing time was orientation-dependent, with moderate to large effects at 0° and 45° (η^2^ = 0.31–0.51), and a small effect at 90° (η^2^ = 0.06).

These findings indicate that printing orientation is the primary determinant of surface hardness in SLA-printed denture base resin. While extended post-curing can enhance hardness, particularly in the 0° orientation, its influence is secondary to the effect of build angle. Specimens printed parallel to the build platform consistently exhibited superior hardness, suggesting more efficient polymer network formation and reduced surface defects compared with angled or vertically printed samples.

## 4. Discussion

This study examined how printing orientation and post-curing time influence the mechanical behaviour of a denture-base resin fabricated by additive manufacturing.

Flexural testing revealed a clear orientation-dependent trend: specimens printed parallel to the build platform (0°) displayed the highest mean flexural strength (FS), with the 0°/30 min subgroup reaching 246.6 MPa, whereas vertically built specimens (90°) were consistently the weakest. This hierarchy (0° > 45° > 90°) mirrors the pattern reported by Shim et al. [12] who attributed the superior 0° values to more homogeneous inter-layer fusion and reduced stair-stepping, and the inferior 90° values to tensile stresses concentrated at inter-layer boundaries.

The existence of two distinct 0° subgroups (A and B) in our design allowed us to probe the influence of part positioning as well as angle. Although both were printed flat, group A with 30 min cure yielded the highest FS, whereas group B after 45 min recorded the lowest value (98.1 MPa). These differences underscore that support placement, exposure to oxygen during irradiation, and heat build-up can all alter the polymer network even at identical nominal angles.

As for the post-curing time for flexural strength, the optimum in our material occurred at 30 min. A similar result has been noted by Bonada et al. [23] as well as by Altarazi et al. [11]. In contrast, Kim et al. [17] found monotonic improvements up to 90 min.

Vickers hardness values increased continuously from 30 to 60 min, in line with the findings of Aati et al. [19], who observed significant increase in hardness, flexural modulus, and fracture toughness when cure time was increased by 20 min. Because microhardness probes only the outer tens of microns, surface-limited post-curing and oxygen inhibition layers dominate its response, whereas FS integrates the entire cross-section; this explains why the two properties did not peak at the same curing time.

Statistical analysis demonstrated that only printing orientation had a significant effect on the mechanical properties, while post-curing time showed no meaningful contribution.

Horizontally built specimens appear to contain less residual monomer at baseline—likely due to their longer effective exposure time per layer—making them more susceptible to subtle reductions in flexural strength when post-curing is extended unnecessarily. In contrast, 90° specimens, whose inter-layer interfaces are inherently weaker, may benefit slightly from additional post-curing that reinforces these bonding regions through modest increases in cross-link density.

The limited improvement in hardness observed in the 90° group, despite prolonged post-curing, can be explained by the interaction between build orientation and polymerisation behaviour in SLA printing. Vertically printed specimens experience a higher cumulative light exposure during fabrication because each layer is cured across the entire height of the specimen. This repeated exposure likely drives a greater degree of polymer conversion during the printing phase itself, leaving fewer unreacted methacrylate groups available for further cross-linking during post-curing. As a result, extended UV irradiation produces only minimal additional network densification, which corresponds to the stable hardness values observed across curing durations. Furthermore, the vertical orientation generates fewer overhangs and shadowed regions, reducing the number of under-polymerised areas that would otherwise benefit from extended curing. Collectively, these factors indicate that vertically printed specimens may approach their maximum achievable cross-link density earlier in the workflow, resulting in limited responsiveness to prolonged post-curing.

From a workflow perspective, selecting printing and post-curing parameters involves balancing production speed, material cost, and mechanical performance. Printing specimens flat on the platform (0° orientation) consistently produced the highest flexural strength, but this configuration also requires greater resin use and more extensive support removal. Post-curing for 30 min—aligned with the manufacturer’s recommendation—yielded flexural strengths comparable to or slightly higher than those obtained after 45 or 60 min, indicating that longer curing does not meaningfully improve resistance to bending. Although surface hardness continued to increase with extended post-curing, the incremental gains between 45 and 60 min were small and may not justify the additional processing time in a high-throughput laboratory environment. Since denture bases are primarily subjected to flexural stresses during mastication, prioritising flexural strength over maximum hardness is generally advisable. However, if enhanced abrasion resistance or polishing durability is clinically important, a 45-min curing time may provide a reasonable compromise between efficiency and mechanical enhancement. Ultimately, laboratories must balance mechanical durability, surface performance, and productivity when determining optimal fabrication protocols for definitive versus interim dentures.

ISO 20795-1 specifies a minimum flexural strength of 65 MPa for denture base polymers. All experimental groups in this study exceeded this threshold, including the weakest configuration (0° Group B, 45-min curing), which remained above 98 MPa. This suggests that, regardless of printing orientation and curing time, the tested SLA-printed material meets basic mechanical requirements for clinical use. Although there is no formal hardness threshold for denture bases, increased surface hardness is associated with better wear resistance and reduced long-term roughening and therefore remains clinically desirable. These comparisons confirm that the material is suitable for denture fabrication, but they also underscore the benefits of optimising printing and curing parameters to enhance performance beyond the minimum acceptable limits.

Fracture behaviour provided additional context for interpreting the flexural strength results. Specimens printed at 0° generally fractured into two large, clean segments, indicating cohesive failure and reflecting the higher strength associated with this orientation. In contrast, 45° specimens showed mixed fracture patterns with partial layer separation, while 90° specimens frequently failed along interlayer boundaries, demonstrating their lower resistance to bending. These patterns confirm that layer orientation and interlayer adhesion strongly influence crack propagation. Clinically, such behaviour is relevant because fractures governed by weak interlayer bonding, as seen in vertically printed specimens, may compromise long-term denture durability under functional loading.

Although both stereolithography (SLA) and digital light processing (DLP) rely on similar photopolymerisation mechanisms, differences in light delivery and layer formation mean that optimal printing orientations are not always comparable across technologies. These variations likely contribute to differences reported in the literature—for example, Yan et al. [14] identified different optimal build angles using DLP than those observed in SLA studies. Thus, recognising the distinctions between additive manufacturing systems is important when interpreting orientation-related results and considering their generalisability.

This study has several limitations. First, flexural strength testing was performed with a relatively small sample size (*n* = 5 per group), which may reduce statistical power. Second, no long-term ageing or thermocycling was conducted, so intraoral degradation processes may alter the mechanical behaviour over time. Third, only one printer, one resin formulation, and one curing unit were evaluated, which limits the generalisability of the findings to other materials and technologies. This study did not assess dimensional accuracy or polymerisation shrinkage; however, these factors are clinically important for denture fit. Future work should evaluate whether the printing parameters identified here also influence the accuracy and adaptation of complete denture bases.

Future research should explore the combined effects of printing orientation with other modifiable parameters, such as layer thickness and support placement. Investigating these interactions may help determine optimal parameter sets for different clinical applications. Additionally, long-term ageing studies and simulated oral environment tests are necessary to evaluate the durability of printed dentures under functional conditions. Such studies would contribute valuable insights into the long-term behaviour and clinical reliability of additively manufactured denture bases.

## 5. Conclusions

Within the limitations of this in vitro study, printing orientation had a significant effect on the mechanical properties of the tested denture base resin, whereas post-curing time showed limited influence. Specimens printed at 0° demonstrated the highest flexural strength, while those printed at 90° exhibited the lowest values. Although flexural strength did not change significantly with extended curing, surface hardness increased progressively from 30 to 60 min.

These findings indicate that flexural strength and hardness respond differently to post-curing duration, and therefore no single curing time simultaneously optimised both properties. For the specific resin and equipment used, printing at 0° consistently produced the strongest specimens, while longer curing primarily enhanced surface hardness. This trade-off is clinically relevant because dentures require high flexural strength to resist fracture and sufficient hardness to minimise wear. These observations apply only to this material–equipment combination and may not be generalisable to other systems.

From a clinical perspective, understanding how build orientation and curing protocols influence mechanical behaviour can support more predictable and efficient fabrication of 3D-printed dentures. Optimising these parameters may contribute to improved durability and reduced need for post-delivery adjustments.

## Figures and Tables

**Table 1:** Flexural strength of 3D-printed denture base resin at different curing times and printing orientations.

Curing Time (min)	Printing Orientation Groups					
	0° (A)	0° (B)	45° (C)	45° (D)	45° (E)	90° (F)
	Flexural Strength (MPa)					
**30**	213.26 (22.25) ^Aa^	145.98 (10.01) ^Ba^	162.04 (7.89) ^Ba^	143.88 (17.46) ^Ba^	137.74 (13.80) ^Ba^	134.98 (10.29) ^Ba^
**45**	208.46 (15.90) ^Aa^	133.4 (20.02) ^Ba^	163.66 (1.7) ^Ba^	146.22 (21.12) ^Ba^	143.14 (17.22) ^Ba^	142.14 (16.02) ^Ba^
**60**	227.58 (11.33) ^Aa^	147 (21.08) ^Ba^	166.16 (7.40) ^Ba^	143.84 (10.31) ^Ba^	157.1 (9.86) ^Ba^	143.36 (8.16) ^Ba^

Within a row, cells with similar (upper case) letters are not significantly different from the control (0° layer orientation). Within a column, cells with similar (lower case) letters are not significantly different from the control (30 min curing time).

**Table 2:** Hardness of 3D-printed denture base resin at different curing times and printing orientations.

Curing Time (min)	Printing Orientation Groups		
	0° (A)	45° (B)	90° (C)
	Vickers Hardness Number (VHN)		
**30**	28.5 (3.32) ^Aa^	16 (1.34) ^Ba^	20.9 (1.37) ^Ba^
**45**	30.1 (3.64) ^Aa^	18.8 (1.46) ^Bb^	20.1 (1.44) ^Ba^
**60**	34 (3.40) ^Ab^	20 (2.04) ^Bb^	20.3 (1.18) ^Ba^

Within a row, cells with similar (upper case) letters are not significantly different from the control (0° layer orientation). Within a column, cells with similar (lower case) letters are not significantly different from the control (30 min curing time).

## Data Availability

The raw data supporting the conclusions of this article will be made available by the authors on request.
